# Bibliometric and visualized analysis of the applications of exosomes for bone regeneration

**DOI:** 10.3389/fcell.2025.1552727

**Published:** 2025-03-17

**Authors:** Shuai Ai, Zhou Xie, Ningdao Li, Runhan Zhao, Xiao Qu, Haining Zhou, Dagang Tang, Jun Zhang, Xiaoji Luo

**Affiliations:** ^1^ Department of Orthopaedic Surgery, The First Affiliated Hospital of Chongqing Medical University, Chongqing Municipal Health Commission Key Laboratory of Musculoskeletal Regeneration and Translational Medicine, Orthopaedic Research Laboratory of Chongqing Medical University, Chongqing, China; ^2^ The First Affiliated Hospital of Chongqing Medical and Pharmaceutical College, Chongqing, China

**Keywords:** exosomes, stem cells, osteogenesis, bone regeneration, bibliometrics

## Abstract

**Background:**

Bone defect, a common orthopedic condition, is characterized by a lengthy and impactful treatment period, posing a considerable challenge in clinical settings. Medical technology has advanced notably, and has effectively treated an increasing number of patients with bone defects. Consequently, there has been an explosion of research articles on bone regeneration, including a substantial number on the application of exosomes. Exosomes, especially those derived from stem cells, have been confirmed to be effective in bone regeneration and have garnered widespread attention in the last decade. Therefore, this study conducted a bibliometric analysis on publications related to the application of exosomes for bone regeneration. The objectives are to explore the development history and research hotspots in this field over the past 10 years, predict future development trends, and provide guidance for subsequent research.

**Methods:**

The Web of Science Core Collection (WoSCC) database was searched for articles related to exosomes and bone regeneration published from 1 January 2014, to 31 December 2023. The collected literature was analyzed using software such as Microsoft Excel, CiteSpace 6.3R1, VOSviewer 1.6.20, and the bibliometric online platform (https://bibliometric.com).

**Results:**

A total of 3,004 articles published by 2,729 institutions from 68 countries were included in this study. The number of articles on the application of exosomes for bone regeneration has increased annually over the last decade. China was the most prolific country in this field, with a total of 1,468 papers; Shanghai Jiao Tong University (China) was the institution with the highest number of publications (117 publications). In terms of authors, Xin Wang, Yi Zhang, and Yang Wang were the three who published the highest number of papers, with 14 papers each. Co-citation analysis revealed that the article published by Valadi H in 2007 has the highest number of co-citations (270 times of quotation). Additionally, most research hotspots focused on the function of exosomes and the mechanism of action. Furthermore, the importance of osteoblast differentiation and angiogenesis in bone regeneration has also garnered significant attention from scholars in this field.

**Conclusion:**

This study reviewed the research achievements on the application of exosomes for bone regeneration over the past 10 years, utilizing bibliometric analysis tools. It visualized the countries, institutions, authors, and journals that have made significant contributions to this field, revealed current research hotspots, and finally explored future development trends.

## Introduction

Bone is the core component of human skeletal tissue and the bearer of movement, providing not only an attachment framework for muscles and other tissues but also protecting internal organs from damage. It plays a crucial role in promoting blood cell production and maintaining the balance of calcium and acid-base homeostasis (Elefteriou, 2018). Bone defects, resulting from trauma or nonunion, remain a major clinical challenge. The annual cost of treating these defects is estimated to be about 45 billion dollars worldwide (Mauffrey et al., 2015; Bharadwaz and Jayasuriya, 2020). Currently, the treatment strategies for clinical bone defects primarily encompass autologous transplantation, allogeneic transplantation, and synthetic biomaterial transplantation (Yu et al., 2022; Baldwin et al., 2019). In recent years, stem cell therapy has emerged as a research focal point and is considered a promising strategy for regenerating bone defects (Tan et al., 2020). Numerous clinical studies have demonstrated that mesenchymal stem cells (MSCs) are safe and effective for treating bone defects (Liebergall et al., 2013; Chen et al., 2016; Castillo-Cardiel et al., 2017; Hernigou et al., 2018a; Hernigou et al., 2018b). Increasing evidence suggests that the beneficial impact of MSCs on tissue repair is primarily due to paracrine effects (e.g., exosomes), which stimulate the activity of tissue-resident recipient cells, rather than through direct differentiation into parenchymal cells to repair or replace damaged tissues (Liang et al., 2014; Zhang et al., 2016). Exosomes are a subclass of membrane-coated extracellular vesicles measuring 30–150 nm in size (Tkach and Théry, 2016). They can exert a variety of biological functions by targeting recipient cells and inducing signaling through receptor-ligand interactions, endocytosis, and/or phagocytosis (Bobrie et al., 2012; Colombo et al., 2014; Hoshino et al., 2015; Shang et al., 2020). Additionally, some studies have indicated that exosomes function by delivering important functional active substances, such as proteins, mRNAs, and microRNAs derived from stem cells (Nikfarjam et al., 2020).

Up to now, the number of corresponding publications on the application of exosomes for bone regeneration has been increasing continuously. However, there has been no a bibliometric analysis to generalize and summarize the articles in this field. Bibliometrics involves the use of mathematical and statistical methods to visually analyze the characteristics of articles published in a specific field, accurately identify the most influential authors, countries, journals, and so on. It also probes into the evolutionary trajectory of this domain and explores the prevalent and prospective focal points within the realm of research. Nowadays, bibliometrics has been widely employed across various subject areas, such as medicine and health, artificial intelligence, and more. Therefore, this study scientifically quantified and quantitatively analyzed the articles published in the WoSCC database from 1 January 2014, to 31 December 2023, on the application of exosomes for bone regeneration, relying on bibliometric analysis tools.

## Data collection and acquisition strategy

**FIGURE 1:**
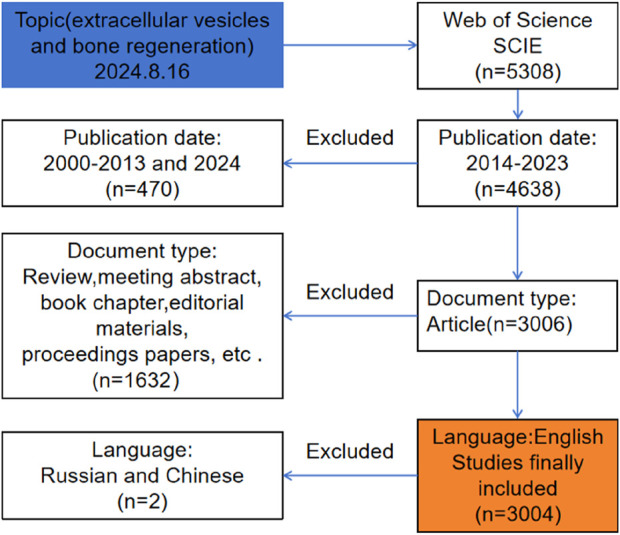
Frame flow diagram of exosomes for bone regeneration from 1 January 2014 to 31 December 2023 based on Web of Science.

This study commenced on 16 August 2024, with all information collected on that day. The WoSCC database encompassed a vast array of publications across various fields and disciplines (Merigó and Yang, 2017), serving as a high-quality digital bibliographic database deemed optimal for bibliometric analysis (Ding and Yang, 2020). Consequently, all information gathered for this study was sourced from the WoSCC database. The search strategy is depicted in Figure 1, and the search formula was as follows: TS=(Exosome OR Exosomes OR “Extracellular vesicle” OR “Extracellular vesicles” OR EVs) AND (Bone OR “Bone Regeneration” OR “Bone Regenerations” OR “Regeneration Bone” OR “Regenerations Bone” OR Osteoconduction). The publication date range was set from 1 January 2014, to 31 December 2023, and the document type was specified as “article.” The language was restricted to English.

### Data analysis and graph production

We extracted the key information from 3,004 articles, including countries, journals, authors, keywords, citations, etc., and imported it into CiteSpace6.3R1, VOSviewer1.6.20, Microsoft Excel, and a bibliometric online analysis platform (http://bibliometric) for processing, analysis, and the production of visual images.

## Results

### Global publication output and citations

**FIGURE 2:**
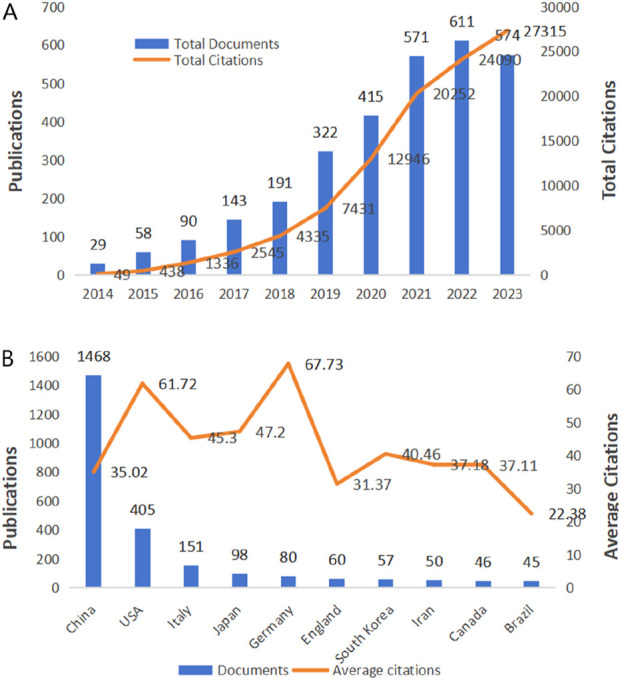
Trends in publications and citations of exosomes for bone regeneration. **(A)** The annual trends of global publications and citations. **(B)** The total citations and average citations of the top ten countries.

We initially searched the WoSCC database for 5,308 studies on the application of exosomes for bone regeneration, and 3,004 articles were ultimately included in our analysis. As seen in Figure 2, there were only 29 publications related to the application of exosomes for bone regeneration worldwide in 2014, whereas the annual output peaked in 2022 with 611 publications. Notably, publications from the past 3 years accounted for 58% (1,756 out of 3,004) of the total, indicating that significant advancements have been made in recent years. Furthermore, the annual citations of all publications from 2014 to 2023 also increased in tandem with the growing number of annual publications, demonstrated that the unique effects of exosomes in bone regeneration have garnered widespread attention from researchers globally.

### Country distribution

**FIGURE 3:**
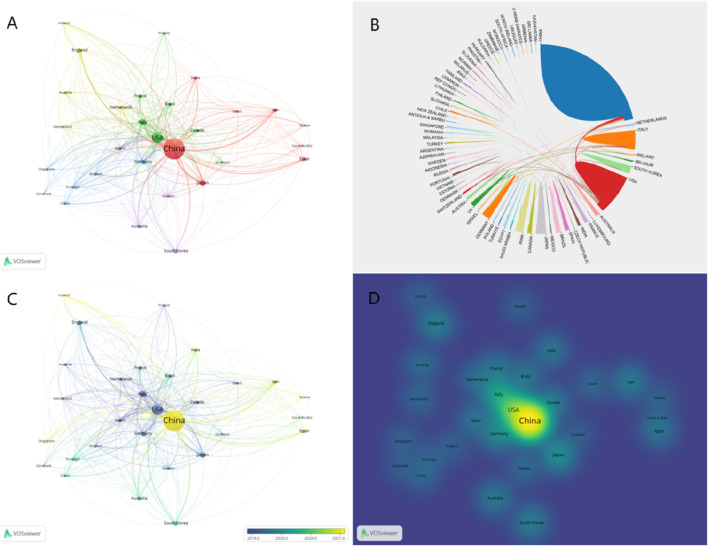
Visualization map of countries involved in exosomes for bone regeneration. **(A)** Collaboration between countries based on VOSviewer. **(B)** Collaboration between countries based on Online Analysis Platform of Literature Metrology. **(C)** Dynamics and trends of countries over time. **(D)** Density map of country distribution of published articles based on VOSviewer.

From 1 January 2014, to 31 December 2023, there were 68 countries and 2,729 institutions published 3,004 publications on the application of exosomes for bone regeneration. The top 10 productive countries, as shown in Table 1, accounted for 82% of all publications (2,460 out of 3,004). Among these countries, China was the most prolific, with 1,468 publications in the field of exosome application for bone regeneration, followed by the United States with 405 publications, Italy with 151, Japan with 98, and Germany with 80. Furthermore, China’s total number of citations was significantly higher than those of other countries. Additionally, Germany had the highest average number of citations (67.73), followed by the United States (61.72) and Japan (47.2), indicating that the studies from these countries were not only of high quality but also widely recognized and frequently cited by researchers in the field. Figure 3 showed that countries around the world closely collaborated with each other in the field of the application of exosomes for bone regeneration. As shown in Figure 3A, the size of each circle represented the number of publications published by a country, and the lines between the circles represented the degree of cooperation between the countries. The results indicated that China cooperated closely with Japan, Australia, and India, while the United States cooperated closely with Italy, Canada, and Belgium (Figures 3A, B). Since 2019, the United States, Italy, and Japan began to augment investment in research on exosomes for bone regeneration, and until 2021, there has been a significant improvement in research for this area in China, Russia, and the United Kingdom (Figure 3C). The density visualization showed that China played an important role in the field of the application of exosomes for bone regeneration (Figure 3D).

**TABLE 1:** The top ten countries that contributed publications on exosomes for the regeneration of bone.

Rank	Country	Documents	Total citations	Average citations
1	China	1,468	51,406	35.02
2	United States	405	24,995	61.72
3	Italy	151	6,840	45.3
4	Japan	98	4,625	47.2
5	Germany	80	5,418	67.73
6	England	60	1882	31.37
7	South Korea	57	2,306	40.46
8	Iran	50	1859	37.18
9	Canada	46	1707	37.11
10	Brazil	45	1,007	22.38

## Institutional distribution

Between 1 January 2014, and 31 December 2023, a total of 2,729 institutions published 3,004 articles on the application of exosomes for bone regeneration. Table 2 displays the top 10 institutions with the highest number of publications. The most prolific institution was Shanghai Jiao Tong University (China, with 117 publications), followed by Central South University (China, with 74 publications) and Nanjing Medical University (China, with 69 publications). Notably, all of the top 10 institutions were from China. From this, we can deduce that China has placed significant emphasis on the study of exosomes for bone regeneration and has invested heavily in this field over the past decade, resulting in a substantial output of research findings.

**TABLE 2:** The top ten institutions that contributed publications on exosomes for regeneration of bone.

Rank	Institutions	Country	Total publications	Total citations	Average citations
1	Shanghai Jiao Tong University	China	117	6,029	51.53
2	Central South University	China	74	1,489	20.12
3	Nanjing Medical University	China	69	3,570	51.74
4	Fudan University	China	64	2,729	42.64
5	Sun Yat Sen University	China	63	2,669	42.37
6	Zhejiang University	China	59	2,526	42.81
7	Huazhong University Sci and Technol	China	59	2,611	44.25
8	Southern Medical University	China	51	1,565	30.69
9	China Medical University	China	50	1,559	31.18
10	Sichuan University	China	42	1,174	27.95

**FIGURE 4:**
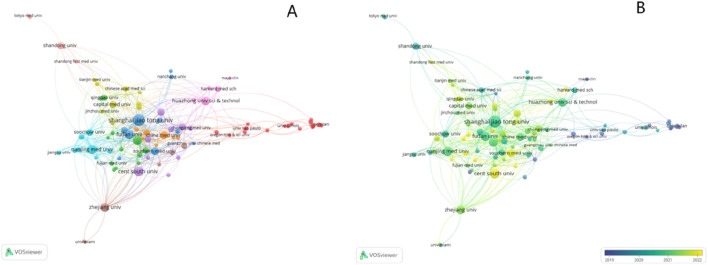
Visualization map of institutions involved in exosomes for bone regeneration. **(A)** Collaboration between institutions involved in exosomes for bone regeneration. **(B)** Dynamics and trends of institutions over time.

As depicted in Figure 4A, institutions worldwide have engaged in close cooperation. For instance, Shanghai Jiao Tong University has collaborated with Harvard Medical School, the University of California, Santa Cruz, and Augusta University. Some institutions embarked on exploring this field earlier, while others have only recently begun to focus on it, such as the University of Pennsylvania and the Third Military Medical University, which initiated research in 2019. In contrast, Southern Medical University and Central South University commenced their involvement in this field in 2022 (refer to Figure 4B).

### Authors and co-cited authors

From 1 January 2014, to 31 December 2023, a total of 15,944 authors participated in the study of exosomes for bone regeneration and published 3,004 relevant papers. The top 10 authors with the highest number of publications in this field were listed in Table 3. Among them, Yan Wang, Yi Zhang, and Xin Wang tied for the first place with the highest number of total publications (14 publications each). Camussi Giovanni, Hui Xie, and Wei Wang followed, sharing second place with 13 publications each. Among these authors, Camussi Giovanni had the highest total citations (74.46) and average citations (74.46), indicating significant achievements and influence in the field of exosome application for bone regeneration.

**TABLE 3:** The top ten authors that contributed publications on exosomes for the regeneration of bone.

Rank	Author	Total publications	Total citations	Average citations
1	Wang, Yan	14	587	41.93
2	Zhang,Yi	14	506	36.14
3	Wang,Xin	14	361	25.79
4	Camussi, Giovanni	13	968	74.46
5	Xie, Hui	13	820	63.08
6	Wang, Wei	13	673	51.77
7	Li,Hui	12	516	43.00
8	Zhang, Lei	12	264	22.00
9	Chen, Lang	11	561	51.00
10	Yang, Yang	11	189	17.18

**FIGURE 5:**
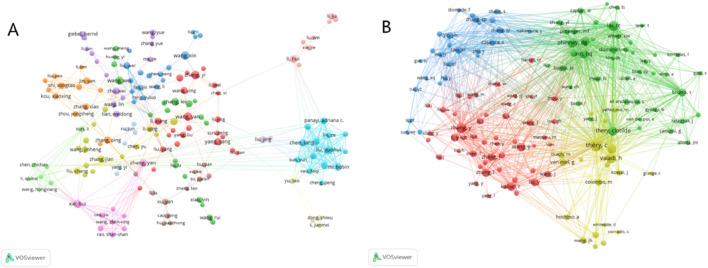
Visualization map of authors and co-cited authors devoted to exosomes for bone regeneration. **(A)** Cooperation network of authors. **(B)** Co-citation network of authors.

Co-authorship analysis can uncover the presence of specific collaborative networks among individual authors. For instanceAUTHORID, authors Yan Zhang, Hui Xie, Shan-shan Rao, and Chun-yuan Chen have collaborated closely in the research on bone regeneration applications (Figure 5A). Co-cited authors are those whose articles are simultaneously cited by the work of another author, thereby forming a co-citation relationship. The higher the frequency of co-citations, the more widely recognized the author’s research is within the field. As depicted in Figure 5B, among the 56,721, co-cited authors, Thery C was the most cited (417 times), followed by Lai RC (288 times) and Zhang Y (288 times).

## Journal distribution and journal co-citation analysis

Regarding academic journals, between 1 January 2014, and 31 December 2023, 703 journals published a total of 3,004 research papers on the application of exosomes for bone regeneration. The top 10 journals were listed in Table 4. From the table, it is evident that the journal with the most publications is *Stem Cell Research and Therapy* (100 articles), followed by the *International Journal of Molecular Sciences* (62 articles), J*ournal of Biomaterials and Tissue Engineering* (52 articles), *Scientific Reports* (34 articles), and *Cells* (33 articles). Additionally, five of the top ten journals originate from the United Kingdom, which may be attributed to the early commencement of academic research and journal development in that country. The remaining two journals are from the United States, two from Switzerland, and one from Australia. It is widely recognized that the impact factor serves as a significant measure to assess the influence of scholarly journals. Among the top 10 academic journals, *Theranostics*, with an impact factor of 12.4, had the highest impact and ranked first in terms of average citations, with an average of 92.77. This indicated its significant influence in the field of exosome application for bone regeneration. Moreover, the Journal Citation Reports (JCR) partition is also a crucial indicator for evaluating journals. Among the top 10 journals, six belong to the Q1 area. The Q1 area indicates that the journal is ranked within the top 25% of its field, representing a collection of high-quality articles from a specific discipline.

**TABLE 4:** Top ten most active journals that published articles onexosomes for the regeneration of bone.

Rank	Journal	Country	JCR	IF	Articles counts	Citations	Average citations
1	Stem Cell Research and Therapy	England	Q1	7.1	100	5,825	58.25
2	International Journal Of Molecular Sciences	Switzerland	Q1	4.9	62	1,111	17.92
3	Journal Of Biomaterials And Tissue Engineering	United States of America	Q4	\	52	16	0.31
4	Scientific Reports	England	Q1	3.8	34	2026	59.59
5	Cells	Switzerland	Q2	5.1	33	843	25.55
6	Journal Of Nanobiotechnology	England	Q1	10.6	32	1,170	36.56
7	Theranostics	Astralia	Q1	12.4	30	2,783	92.77
8	Journal Of Cellular And Molecular Medicine	England	Q2	4.3	29	1,187	40.93
9	Stem Cells International	England	Q2	3.8	29	904	31.17
10	Plos One	United States of America	Q1	2.9	28	1,634	58.36

**FIGURE 6:**
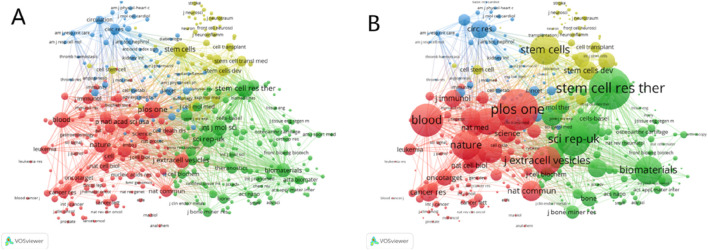
**(A)** Visualization map of co-cited journals of studies related to exosomes for bone regeneration. **(B)** The partial enlarged drawing of the main co-cited journals.

Journal co-citation is a co-citation relationship that is established with journals as the fundamental unit, reflecting the influence of journals within specific fields. As depicted in Figure 6, the most cited journal is *Stem Cell Research and Therapy* (2,501 times), followed by *Plos One* (2,482 times) and *SCI REP-UK* (1965 times).

**FIGURE 7:**
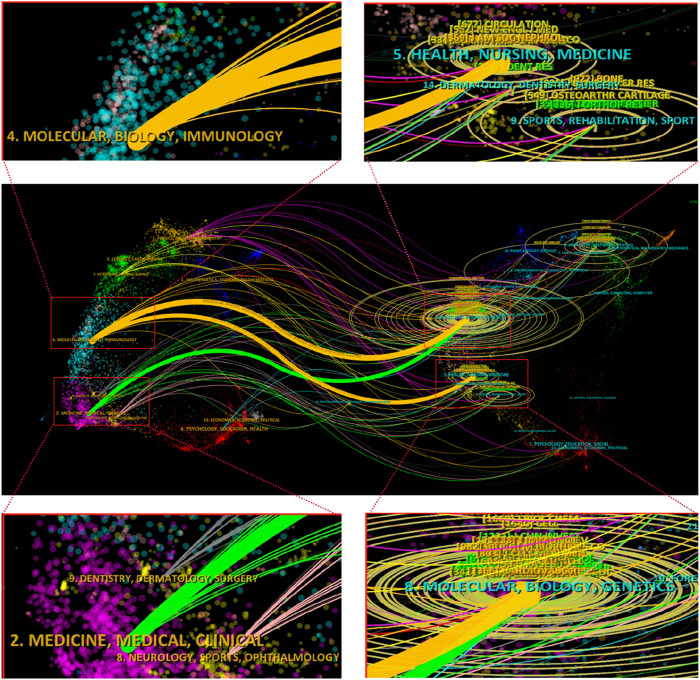
The dual-map overlay of journals related to exosomes for bone regeneration.

By employing the journal double graph overlay produced by Cite Space software, we can examine the distribution of relationships among journals. As depicted in Figure 7, the right side represents the collection of cited journals, predominantly basic theory journals. Conversely, the left side depicts the citing journals, which are primarily from the Frontier of knowledge. The color path represented the citation relationship between journals. Figure 7 illustrated three significant pathways: one green and two yellow. The green pathway indicates that papers published in journals such as MEDICINE, MEDICAL, and CLINICAL frequently cite articles from journals like MOLECULAR, BIOLOGY, and GENETICS. The upper yellow pathway shows that papers in journals such as MOLECULAR, BIOLOGY, and IMMUNOLOGY consistently reference articles from MOLECULAR, BIOLOGY, and GENETICS. The lower yellow pathway demonstrates that papers in MOLECULAR, BIOLOGY, and IMMUNOLOGY journals often cite articles from journals such as HEALTH, NURSING, and MEDICINE.

### References co-cited analysis

Reference co-citation analysis is crucial for tracing scientific frontiers and research foundations (Zhang et al., 2022a). It refers to the phenomenon where two or more documents are simultaneously cited by one or more subsequent papers, thereby forming a co-citation relationship. Co-citation in literature indicates the strength of the correlation between articles. Through co-citation analysis, we can explore not only the development and evolutionary dynamics of the discipline but also identify frequently cited literature within a specific field, thereby revealing important theories and research findings in that area. Table 5 listed the ten most frequently cited references. The top 10 papers were all cited at least 111 times. Among these papers, the one published by Valadi H in 2007 was the most cited (270 citations). The second and third most cited articles were both published by Théry C, with the second in 2006 (207 citations) and the third in 2018 (206 citations).

**TABLE 5:** The top 10 co-cited references related to exosomes for the regeneration of bone.

Rank	Auther	Citationgs	Title	Journal	Year
1	Valadi,H	270	Exosome-mediated transfer of mRNAs and microRNAs is a novel mechanism of genetic exchange between cells	NATURE CELL BIOLOGY	2007
2	Théry,C	207	Isolation and characterization of exosomes from cell culture supernatants and biological fluids	CURRENT PROTOCOLS IN CELL BIOLOGY	2006
3	Théry,C	206	Minimal information for studies of extracellular vesicles 2018 (MISEV 2018): a position statement of the International Society for Extracellular Vesicles and update of the MISEV2014 guidelines	JOURNAL OF EXTRACELLULAR VESICLES	2018
4	Raposo, G	186	Extracellular vesicles: Exosomes, microvesicles, and friends	JOURNAL OF CELL BIOLOGY	2013
5	Kalluri,R	176	The biology, function, and biomedical applications of exosomes	SCIENCE	2020
6	Domonici,M	153	Minimal criteria for defining multipotent mesenchymal stromal cells. The International Society for Cellular Therapy position statement	CYTOTHERAPY	2016
7	Phinney, DG	148	Concise Review: MSC-Derived Exosomes for Cell-Free Therapy	STEM CELLS	2017
8	Lai, RC	143	Exosome secreted by MSC reduces myocardial ischemia/reperfusion injury	STEM CELL RESEARCH	2010
9	Colombo,M	121	Biogenesis, Secretion, and Intercellular Interactions of Exosomes and Other Extracellular Vesicles	ANNUAL REVIEW OF CELL AND DEVELOPMENTAL BIOLOGY	2014
10	Tkach,M	111	Communication by Extracellular Vesicles: Where We Are and Where We Need to Go	CELL	2016

**FIGURE 8:**
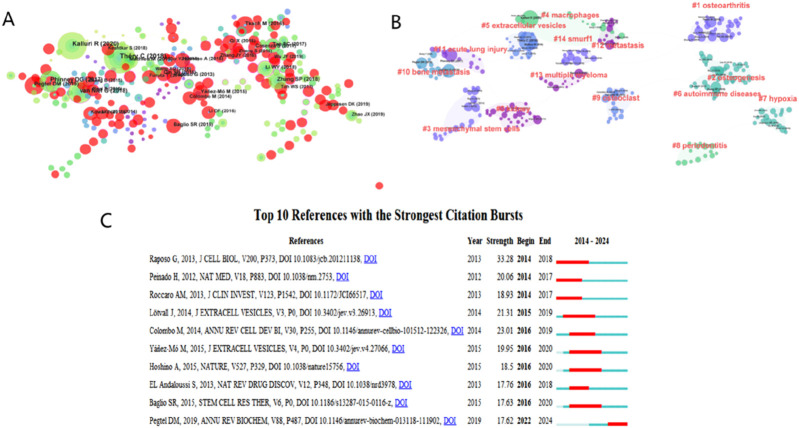
**(A)** Visualization map of co-cited references analysis based on CiteSpace. **(B)** Cluster analysis of co-cited references based on CiteSpace. **(C)** Visualization map of top ten references with the strongest citation bursts related to exosomes for the regeneration of bone based on CiteSpace.

Additionally, we utilized CiteSpace software to create a visual network representation of co-cited articles and a co-citation cluster map to intuitively display the distribution relationship among co-cited articles. Figure 8A depicted the visualization of co-cited articles, with the size of the circle indicating the number of co-citations. Figure 8B illustrated the clusters of co-cited articles. A total of 15 clusters were formed by the co-cited articles, and the top 10 clusters were “#0 kidney”, “#1 osteoarthritis”, “#2 osteogenesis”, “# mesenchymal stem cells”, “#4 macrophages”, “#5 extracellular vesicles”, “#6 autoimmune diseases”, “#7 hypoxia”, “#8 periodontitis” and “#9 osteoclast”. What’s more, CiteSpace software was used to construct the strongest citation explosion graph of the top 10 references. As depicted in Figure 8C, the lines delineate the time frame from 2014 to 2024, with the red lines marking the period during which citations experienced a surge. The top 10 references exhibited an intensity ranging from 17.62 to 33.28 and a duration of 3–5 years. The reference with the strongest citation burst was a review published by Raposo G in 2013, which focused on the characteristics of exosomes and the mechanisms by which exosomes behave, anchor and function (Raposo and Stoorvogel, 2013). It is evident that the article experienced a surge in citations from 2014 to 2018, suggesting that numerous related studies were published during this period. The article that experienced the second highest citation burst was published by Colombo M in 2014. It explored the effect of exosomes on tumorigenesis and indicated that exosomes played a supportive role in tumor growth and metastasis, offering a new perspective for tumor treatment (Colombo et al., 2014).

### Keywords co-occurrence analysis

**FIGURE 9:**
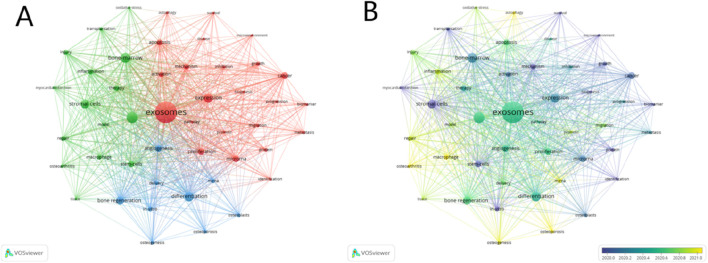
Mapping of keywords in studies on exosomes for the regeneration of bone based on VOSviewer. **(A)** Network visualization of keywords. **(B)** Chronological order of keywords.

Keywords are an important part of articles and represent the research focus and core content of papers. The analysis of keywords can assist in summarizing the evolution of research hotspots in specific fields and exploring future research directions. Therefore, we analyzed the keywords of the 3,004 papers collected and visualized the map using VOSviewer software. A total of 7,763 keywords were extracted and analyzed in this study. As shown in Figure 9A, 47 keywords appeared in the figure (with a minimum occurrence frequency of 60 times). The top ten keywords with the highest frequency were “exosomes” (1888), “mesenchymal stem cell” (632), “bone-marrow” (509), “differentiation” (473), “expression” (458), “stromal cells” (412), “bone regeneration” (363), “cancer” (296), “angiogenesis” (278) and “microrna” (271). All of the keywords in Figure 9A were primarily divided into three clusters, colored red, green, and blue respectively. The red cluster, comprising a total of 23 keywords, primarily represented basic biology research, with representative keywords such as “expression”, “protein”, and “microRNA”. The green cluster, comprising a total of 15 keywords, primarily represented clinical practice research. The representative keywords included “inflammation”, “injury”, and “therapy”. The blue cluster, with 9 keywords in total, represented other studies related to the application of exosomes for bone regeneration. The representative keywords were “angiogenesis”, “differentiation”, and “bone regeneration”. As exploration in specific fields deepens, new research hotspots will generate novel keywords. As depicted in Figure 9B, keywords are color-coded to signify particular time periods. The most recent keywords, indicated by yellow, reveals that “osteoporosis,” “osteogenesis,” and “macrophage” have been research focal points in recent years within the domain of exosome application for bone regeneration.

## Discussion

In this study,we conducted a systematic bibliometric analysis to intuitively trace the evolutionary course of exosome application in bone regeneration over the past 10 years and summarized major achievements in this field, which are beneficial for identifying new research hotspots. For instance, exosomes secreted by MSCs played a crucial role in bone regeneration (Zeng and Xie, 2022). MSCs are stromal cells that are plastic-adherent and able to differentiate into osteoblasts, chondroblasts, and adipocytes (Jin and Lee, 2018). They were first found in bone marrow and have been used to promote bone healing for approximately 20 years (Hernigou et al., 1997). In recent years, an increasing number of scholars have shown interest in studying exosomes derived from various mesenchymal stem cells (MSCs), such as adipose-derived MSCs, bone marrow MSCs, and others, what followed was the application of exosomes in bone regeneration. Although many achievements have been made in understanding exosomes in bone regeneration (Li et al., 2024), the mechanisms and active components have not been fully clarified and require further exploration. Besides, some scholars found that exosomes played a significant role in the initiation, development, evolution, and metastasis of tumors (Yang et al., 2024), which may offer new insights for the exploration of tumor treatment.

China emerged as the most prolific country in this field, publishing 49% of all papers (1,468 out of 3,004) and accumulating the highest total citations (51,406), yet with a relatively low average citation count (35.02 average citations). This suggested that while China’s research in this area has entered a period of rapid development and some achievements have been made, it remains in the exploratory stage. Future research will require more high-quality papers. The United States ranked second in terms of total number of publications and total citations, with an average citation of 61.72. This demonstrated that the United States had a high level of scientific research, published a large number of high-quality papers and made significant contributions to the study of exosome application in bone regeneration. Despite publishing only 80 papers, Germany had the highest average number of citations (67.73 average citations), indicating that the country emphasized the quality of papers, aimed for excellence in academic research, and that the study achievements on the application of exosomes for bone regeneration were widely recognized within the industry. Besides, the contribution of scientific research achievements of institutions reflects the level of scientific research development of countries or regions. It is significant that the top 10 institutions that published the most papers on the study of exosomes and bone regeneration were all Chinese universities and research institutes, indicating that China has made tremendous improvements in recent years in the development of university education and scientific research, with an increasing number of universities and institutes now having the ability and conditions to produce more scientific research results. Shanghai Jiao Tong University (China) was the institution that produced the most scientific research results, published 117 papers on the application of exosomes for bone regeneration. This institution focused on the application of various biomaterials or bioactive substances in the regeneration of bone defects. Their recent study found that the electrophysiological microenvironment and bioactive irons have a synergistic effect in bone regeneration (Deng et al., 2024), this seems to be a novel and interesting research direction.

There were specific journal distributions for different fields of study. Bibliometric analysis aids us in identifying the primary journals where papers are most frequently published within a specific field, uncovering the most productive and influential journals, and offering guidance for future research in the related area (Zhang et al., 2022b). Among the top 10 journals ranked based on the number of published papers, *Stem Cell Research and Therapy* (IF = 7.1) boasted the highest number of published articles (100 publications) and the highest total number of citations (5,825 total citations). Additionally, its average number of citations (58.25 average citations) was notably high, indicating that this journal is highly popular within the field of exosome application for bone regeneration. Most scholars prefer to publish or consult relevant papers in this journal. Additionally, *Theranostics* holds the highest impact factor (IF = 12.4) and the highest average citations (Average Citations = 92.77), indicating that this journal features high-quality papers, representing the pinnacle of paper quality in this field, and that papers published in this journal are more likely to receive further attention and citations from scholars. This journal was mainly devoted to experimental medical research. Recently, Su Ni et al. demonstrated that aspirin and mineral-particle coated scaffolds offered a promising therapy for repairing critical-size bone defects through immune regulation and highlight the importance of optimizing mineral particle and aspirin dosages to achieve robust bone healing (Su et al., 2023).

According to the authors, Yan Wang, Yi Zhang, and Xin Wang were the three most prolific contributors in the field of exosome application for bone regeneration, have each published 14 papers. Among the three authors, Yan Wang had the highest total and average citations, with 587 total citations and an average of 41.93 citations. His team was working on a series of studies on the bio-active molecules and mechanisms of exosomes. In recent years, they found that exosomes produced by bone-marrow MSCs significantly reduced the apoptosis rate and reactive oxygen species (ROS) production of cardiac stem cells in response to oxidative stress (Wang et al., 2018). Aditionally, The author Giovanni Camussi, while not the most prolific, had the highest total and average citations (968 total citations and 74.46 average citations), indicating that his papers exerted enormous influence. What’s more, Giovanni Camussi and his team explored the exosomes derived from bone-marrow and adipose-tissue MSCs. They analyzed the exosomes of two different vectors, ADSC-EV (adipose-derived MSCs-Extracellular vesicles) and BMSC-EV (bone-marrow MSCs-Extracellular vesicles), and found that the molecular agents present in ADSC-EV were highly correlated with angiogenesis, while the molecules expressed in BNSC-EV were preferentially involved in cell proliferation. What’s more, ADSC-EV and BMSC-EV have been demonstrated to have beneficial effects on cells involved in skin injury healing, such as fibroblasts, keratinocytes, and endothelial cells (Pomatto et al., 2021).

Among the top 10 most cited literature, the most cited was “*Exosome-mediated transfer of mRNAs and microRNAs is a novel mechanism of genetic exchange between cells”* (270 citations). This article was a basic study on exosomes conducted by Hadi Valadi and his colleagues. They concluded that exosomes contain both mRNA and microRNA, which can be delivered to another cell and function in this new location, and they called this RNA as “exosomal shuttle RNA” (esRNA) (Valadi et al., 2007). Analysis of the co-cited references indicated that at least 270 subsequent studies on exosomes have referenced this article. These include research explored the application of adipose stem cell exosomes in the immune regulation of asthma patients (Jung et al., 2024), and studied on the use of mesenchymal stem cell exosomes for the treatment of osteoarthritis (Vadhan et al., 2024), etc. This indicates that basic theoretical research is the foundation for the functional discovery and clinical application of exosomes, fully illustrating the importance of basic theoretical research.

The keywords with high frequency in keywords analysis were “exosomes” (1888), “mesenchymal stem cell” (632), “bone-marrow” (509), “differentiation” (473), “expression” (458), “stromal cells” (412), “bone regeneration” (363), “cancer” (296), “angiogenesis” (278), “microrna” (271). Regarding the mechanisms of exosomes action in bone regeneration, there is still no consensus. Exosomes may enhance the expression and secretion of specific proteins in MSCs, thereby accelerate osteogenic differentiation and bone metabolism, ultimately achieve the goal of promoting bone regeneration. In addition, bone regeneration was deeply linked to angiogenesis. Jacopo Pizzicannella et al. found that the expression of osteogenic markers increased when exosomes inducing bone regeneration, which was also accompanied with the increase of the expression of angiogenesis factors, and histology also demonstrated the activation of bone regeneration and vascularization in this process (Pizzicannella et al., 2019). “Osteogenesis”, “osteoporosis”, and “macrophage” were the research hotspots in recent years. Osteogenic differentiation is a critical step in bone regeneration, one study of Mengmeng L showed that miR-324-containing exosomes released by mature osteoclasts played an significant role in the regulation of osteogenic differentiation, and that miR-324-rich exosomes-modified scaffolds displayed the highest bone regeneration capacity in mouse models of skull defects, this proved that exosomes played an important role in osteogenic differentiation (Liang et al., 2021). In recent years, the treatment of osteoporotic bone defects received widespread attention. Osteoporosis is typified by atypical activity of osteoclasts and compromised osteogenic capacity in bone-marrow MSCs, thereby disrupting the equilibrium of skeletal homeostasis. Clinically, this disorder is evidenced by a diminished bone mineral density, alterations in the architecture of bone trabeculae, and a protracted duration for bone repair processes (Cummings and Melton, 2002). Meng FY et al. found that exosomes derived from young plasma (Y-Exos) alleviated osteoporosis and exhibited significant enhancement in BMSC proliferation, migration and osteogenic differentiation compared with those derived from aged plasma. In addition, the Y-Exos significantly reduced osteoporosis and promoted bone regeneration. Furthermore, thier study found that exosomal miR-217–5p is a key contributor to the osteoprotective effects of young plasma-derived exosomes (Meng et al., 2024). This provides a potential strategy for advancing clinical treatment of osteoporotic bone defects. In addition, the positive role of macrophage-derived exosomes in bone regeneration has also received widespread attention. A retrospective study by Daneshvar Alireza and his colleagues confirmed that exosomes derived from macraphage-2 macrophages can effectively promote bone regeneration and vascularization in animal models of bone defects, and they observed positive effects on cell proliferation, migration, osteogenesis and angiogenesis *in vitro* studies, therefore, they concluded that M2 macrophage-derived exosomes exhibited significant potential to promote bone regeneration (Daneshvar et al., 2024).

In recent years, exosomes have remained highly popular in bone regeneration, with a wealth of innovative research emerging. The latest research by Yi-kun Zhou and his team indicated that H_2_S pretreatment can promote the polarization of M2 macrophages to increase the osteogenic ability of MSCs, and this reinforcing effect is achieved through exosomes derived from M2 macrophages (Zhou et al., 2024). They found that exosomes secreted by pretreated M2 macrophages can promote the osteogenic differentiation of MSCs *in vitro* and *in vivo*, and this was verified in a cranial defect model. Further research found that exosomes derived from pretreated M2 macrophages were more easily internalized by MSCs, and H2S pretreatment promoted the enrichment of the exosomal protein moesin, which, upon internalization, activated the β-catenin signaling pathway in MSCs to promote bone regeneration. In addition, the application of metal-organic frameworks and metal ions in bone regeneration has become a hot topic in recent years and is a promising strategy for bone tissue regeneration. Bone regeneration is a complex process in which various divalent cations function, including Ca2+, Cu2+, Mg2+, etc. Jun Z and his team described a novel material designed in a study, the electrospun short nanofibrous sponges (3D-NS) functionalized with phosphorus dendrons (3D-NS@PD), which can dynamically capture free copper ions at multiple sites, maintain the concentration of free copper ions at a certain level, and prove *in vitro* and *in vivo* and animal bone defect models that this material effectively promotes bone regeneration by managing the concentration of copper ions (Zhang et al., 2023). Furthermore, Kang Yue et al. creatively combined human adipose-derived stem cell-derived exosomes with metal magnesium organic frameworks (MOFs) and gallic acid (GA) to design and synthesize a cell-free PLGA/Mg-GA MOF scaffold (PLGA/Exo-Mg-GA MOF), utilizing the advantages of hADSCs-Exos, Mg^2+^, and gallic acid (GA) to construct a unique nanoscale interface, stimulating the release of Mg^2+^ and exosomes, creating a pro-angiogenic biochemical microenvironment, and simultaneously enhancing the osteogenic ability of bone marrow MSCs (Kang et al., 2022). Their studies showed that the PLGA/Exo-Mg-GA2 scaffold has great potential in promoting new bone growth *in vivo*. Antler blood (ALB) is a valuable traditional Chinese medicine known for its potent regenerative properties. To investigate the relationship between ALB and exosomes derived from bone-marrow mesenchymal stem cells (BMSC-Exos), Zuo Renjie and colleagues extracted the primary components of ALB and utilized them to pretreat BMSCs, resulting in specialized exosomes named ALB-Exos. The osteogenic and angiogenic potential of these exosomes was then assessed through experimental studies. Their studies showed that ALB and BMSC-Exos separately exhibit significant promotion of bone and vascular formation, and enhance the expression of BMP-2 (Bone morphogenetic protein 2), RUNX2 (Runt-related transcription factor 2), and ALP (Alkaline Phosphatase), with ALB-Exos showing the strongest performance in these functions (Zuo et al., 2024). In addition, they pointed out that the ability of ALB to enhance the osteogenic and angiogenic potential of BMSC-derived exosomes may be related to the upregulation of miR-21–5p. Abave all, these studies indicated that there is still great potential for the application of exosomes in bone regeneration, including these derived from MSCs or others, which awaits further exploration by scholars in this field.

Of course, this study has some limitations. First, although WoSCC was recognized as the most reliable and most commonly used database for bibliometric analysis, it does not covered all academic papers published, and the data collected in this study are only from the WoSCC database, so the data obtained was not as so comprehensive. Second, all the information in this study was read by software tools, which inevitably leads to potential bias. Finally, publications of 2024 were not included in our study because the data was incomplete at the time we conduct this study.

## Conclusion

Relying on Bibliometric analysis tools, We systematically analysed the research results related to the application of exosomes for bone regeneration published from 1 January 2014 to 31 December 2023, identified countries, institutions, authors and journals that have made significant contributions to this field in the past 10 years, and furthermore summarized some significant research results. In addition, this study confirmed the positive role of exosomes in bone regeneration, and pointed out that exosomes have great potential in not only bone regeneration but also in the improvement of cardiovascular function, tissue repair, tumor treatment and so on.

## Data Availability

The raw data supporting the conclusions of this article will be made available by the authors, without undue reservation.
